# *HYAL2* methylation in peripheral blood as a potential marker for the detection of pancreatic cancer: a case control study

**DOI:** 10.18632/oncotarget.18757

**Published:** 2017-06-27

**Authors:** Sarah Schott, Rongxi Yang, Sarah Stöcker, Federico Canzian, Nathalia Giese, Peter Bugert, Frank Bergmann, Oliver Strobel, Thilo Hackert, Christof Sohn, Barbara Burwinkel

**Affiliations:** ^1^ Molecular Biology of Breast Cancer, Department of Gynecology and Obstetrics, University of Heidelberg, 69120 Heidelberg, Germany; ^2^ Department of Gynecology and Obstetrics, University Women's Clinic, 69120 Heidelberg, Germany; ^3^ German Cancer Consortium (DKTK), NCT Heidelberg and German Cancer Research Center (DKFZ), 69120 Heidelberg, Germany; ^4^ Molecular Epidemiology (C080), German Cancer Research Center (DKFZ), 69120 Heidelberg, Germany; ^5^ Genomic Epidemiology Group (C055), German Cancer Research Center (DKFZ), 69120 Heidelberg, Germany; ^6^ Department of General, Visceral and Transplantation Surgery, Heidelberg University Hospital, 69120 Heidelberg, Germany; ^7^ Institute of Transfusion Medicine and Immunology, Medical Faculty Mannheim, University of Heidelberg, German Red Cross Blood Service Baden-Württemberg – Hessen, 68167 Mannheim, Germany; ^8^ Institute of Pathology, University Hospital Heidelberg, 69120 Heidelberg, Germany

**Keywords:** HYAL2, methylation, pancreatic cancer, early detection, marker

## Abstract

Pancreatic ductal adenocarcinoma (PDAC) is a highly lethal malignancy which is mostly diagnosed in advanced and inoperable stages though surgery remains the only curable therapeutic approach. Early detection markers are urgently needed to improve diagnosis. Altered hyaluronoglucosaminidase 2 gene (*HYAL2*) DNA methylation in peripheral blood is known to be associated with malignancy at early stage but has not been evaluated in PDAC patients. This study evaluates the association between blood-based *HYAL2* methylation and PDAC by a case-control study with 191 controls and 82 PDAC patients. Decreased methylation of all four investigated *HYAL2* methylation sites showed highly significant association with PDAC (odds ratio (ORs) per −10% methylation ranging from 2.03 to 12.74, depending on the specific CpG site, *p <* 0.0001 for all). *HYAL2* methylation sites were also distinguishable between stage I&II PDAC (61 subjects) and controls (ORs per-10% methylation from 3.17 - 23.04, *p <* 0.0001 for all). Thus, *HYAL2* methylation level enabled a very good discrimination of PDAC cases from healthy controls (area under curve (AUC) = 0.92, 95% Confidence interval (C.I.): 0.88 - 0.96), and was also powerful for the detection of PDAC at stage I&II (AUC = 0.93, 95% C.I.: 0.89 - 0.98). Moreover, the blood-based *HYAL2* methylation pattern was similar among PDAC patients with differential clinical characteristics, and showed no correlation with the overall survival of PDAC patients. Our study reveals a strong association between decreased *HYAL2* methylation in peripheral blood and PDAC, and provides a promising blood-based marker for the detection of PDAC.

## INTRODUCTION

Pancreatic ductal adenocarcinoma (PDAC) is a highly malignant cancer and the fourth leading course of cancer-related mortality with 40,000 deaths in Europe each year [1]. The therapeutic options are devastating and surgery remains the main curative treatment approach for only 20% of patients, whereas the other patients are not operable. Even in the 20% patients who could be treated by radical pancreatico-duonodectomy, the 5-year survival is also just about 30% and chemotherapy had only limited improvement in survival [2–3]. Overall, 10-year survival of PDAC patients is only 1.1% [4]. Since early detection strategies are missing, most patients present clinically with a progressed and incurable disease which has very limited curative therapeutic approaches.

Major risk factors for PDAC are known to be chronic pancreatitis and environmental risk factors such as tobacco use, high caloric diet and alcohol as well as inherited cancer susceptibility syndromes in up to 10% of the cases [5]. Multiple genes that are associated with well-known hereditary cancer syndromes such as *BRCA1* and *BRCA*2, *PALB2*, *CDKN2A*, *ATM* and the DNA mismatch repair genes were found to be associated with pancreatic ductal adenocarcinoma as well [6]. Recently, inherited mutations in correlation with PDAC have gained focused attention, and a pathogenic *BRCA1* and *BRCA*2 mutation was identified in a large cohort in 4.6% among pancreatic ductal adenocarcinoma patients [6]. Nonetheless, those risk stratifications attempts have not yet been implemented in daily clinic to improve diagnostic tools.

There are several approaches to identify new biomarkers and to classify pancreatic cancer carriers from healthy persons. DNA methylation is described as one of the earliest and most common events in the process of cancer development, affecting control of gene transcription and the architecture of the cell nucleus [7, 8]. Recent literature has reported the correlation between aberrant methylation of DNA from peripheral blood and multiple cancers, such as breast, ovarian, head and neck, as well as bladder cancer [9–20].

Hyaluronidases (HYALs) are key regulators of hyaluronan (HA) metabolism, and HYAL2 is known to be expressed in somatic tissue and blood cells and to initiate HA degradation [21], whereas HYAL1 expression in tumor tissue was recently found to be associated with endometrial carcinoma aggressiveness and described as an independent prognostic factor for early disease recurrence [22]. At the somatic level, overexpression of HYAL2 is correlated with a higher occurrence of metastasis and shorter survival of triple negative breast cancer [18]. Whole exome sequencing discovered *HYAL2* mutations in the recurrent B-cell lymphoma [23]. Furthermore, the methylation level of *HYAL2* in tumor tissue has been shown to predict overall and progression-free survival in colorectal cancer [24]. Decreased methylation of *HYAL2* has been reported in the peripheral blood DNA of breast cancer patients and head and neck cancer patients [11, 25].

To investigate the association between blood-based *HYAL2* methylation and PDAC, we hereby conducted a case-control study and analysed the *HYAL2* methylation in PDAC patients with various clinical characteristics (Table 1) and healthy control individuals.

**Table 1:** The clinical characteristics of PDAC patients

Clinical characteristics	Group	N (%)	Median of age
**Tumour size**	T1	3 (3.7%)	64.2
	T2	1 (1.2%)	63.1
	T3	62 (75.6%)	63.2
	T4	4 (4.9%)	50.7
	Unknown	12 (14.6%)	68.5
**Lymph node involvement**	pN0	16 (19.5%)	61.5
	pN1	54 (65.9%)	63.2
	Unknown	12 (14.6%)	68.5
**Status of distant metastasis**	M0	66 (80.5%)	62.8
	M1	12 (14.6%)	69.1
	Unknown	4 (4.9%)	67.6
**Tumour stage**	Stage I	4 (4.9%)	63.7
	Stage II	57 (69.5%)	62.9
	Stage III	4 (4.9%)	50.7
	Stage IV	12 (14.6%)	69.1
	Unknown	5 (6.1%)	68.8
**Grading**	Grade 1	2 (2.4%)	68.5
	Grade 2	33 (40.2%)	64.4
	Grade 3	22 (26.8%)	60.5
	Unknown	25 (30.5%)	63.6
**Gender**	Male	49 (59.8%)	63.1
	Female	33 (40.2%)	64.4

## RESULTS

### Decreased *HYAL2* methylation is associated with PDAC

**Figure 1:**
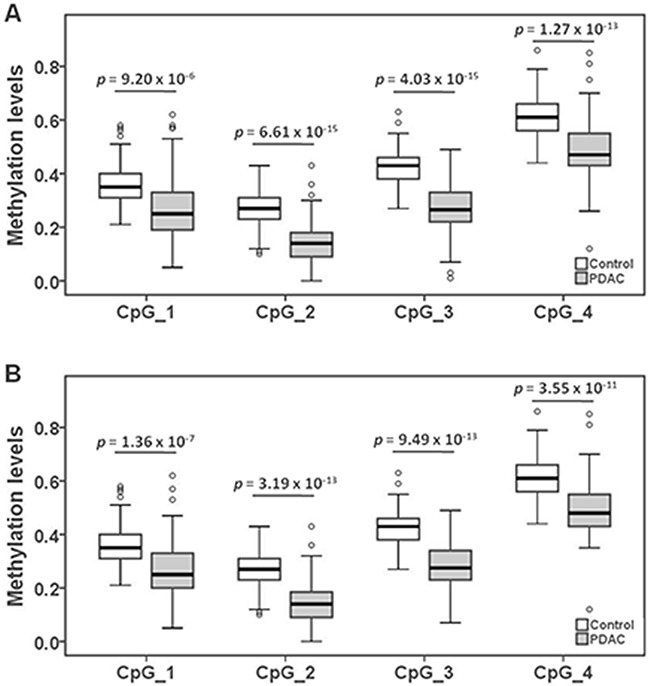
The association between decreased *HYAL2* methylation in peripheral blood DNA and PDAC **(A)** The box plots show the distribution of *HYAL2* methylation levels in PDAC cases and controls. **(B)** The box plots show the distribution of *HYAL2* methylation levels in Stage I&II PDAC cases and controls. The *p*-values were calculated by logistic regression adjusted for age and gender. The circles indicate outliers.

*HYAL2* methylation in peripheral blood DNA associated to PDAC was evaluated using the HYAL2-A amplicon [25] harboring four CpG sites (CpG_1, CpG_2, CpG_3, CpG_4) for analyses with Sequenom MALDI-TOF (matrix-assisted laser desorption ionization time-of-flight) mass spectrometry. The HYAL2_CpG_3 site showed the most significantly lower methylation levels in PDAC cases than in controls (PDAC cases: median = 0.27 (inter quartile range (IQR) = 0.22-0.33); controls: median = 0.43 (IQR = 0.38-0.46); odds ratio (OR) per −10% methylation = 12.74, 95% C.I. = 6.75-24.04, *p =* 4.03 × 10^−15^ by logistic regression adjusted for age and gender, Figure 1A and Table 2). Methylation levels of the other three CpG loci also showed significantly lower levels among the PDAC cases than the controls (all OR per −10% methylation > 2.00, *p* < 1.00 × 10^−5^ by logistic regression adjusted for age and gender, Figure 1A and Table 2). The methylation levels of the other three CpG loci in HYAL2-A amplicon [25] were also strongly correlated with the methylation level of HYAL2_CpG_3 (all Spearman rho > 0.65, *p* < 4.60 × 10^−34^, Table 2).

**Table 2:** Methylation difference of *HYAL2* comparing PDAC cases and controls

CpG sites	Differences in methylation levels				Correlations to HYAL2_CpG_3	
	Controls median (IQR)	PDAC cases median (IQR)	OR (95 % CI) * per −10% methylation	*p*-value *	Spearman rho	*p*-value
**HYAL2_CpG_1**	0.35 (0.31-0.40)	0.25 (0.19-0.34)	2.03 (1.49-2.78)	**9.20E-06**	0.65	4.55E-34
**HYAL2_CpG_2**	0.27 (0.23-0.31)	0.14 (0.09-0.18)	8.50 (4.96-14.57)	**6.61E-15**	0.82	1.09E-67
**HYAL2_CpG_3**	0.43 (0.38-0.46)	0.27 (0.22-0.33)	12.74 (6.75-24.04)	**4.03E-15**	1.00	-
**HYAL2_CpG_4**	0.61 (0.56-0.66)	0.47 (0.43-0.55)	5.10 (3.32-7.85)	**1.27E-13**	0.82	1.79E-66

* Logistic regression, adjusted for age and gender

### Decreased *HYAL2* methylation is associated with PDAC at stage I&II

Since the PDAC patients recruited in this study are part of a surgical collective, more early and resectable cases (Stage I&II) were included than typical for the general PDAC population. 60 out of the 82 PDAC are at the stage I and stage II. Compared to the healthy controls, the status of *HYAL2* methylation was also significantly decreased in PDAC cases at relatively early stage. The methylation status of all *HYAL2* CpG sites was also correlated with stage I&II PDAC as shown by the OR > 3.17 (*p* < 1.40 × 10^−7^ by logistic regression adjusted for age and gender, Figure 1B and Table 3). Same as the PDAC in general, the most altered methylation for stage I&II PDAC compared to controls was detected in the HYAL2_CpG_3 site (OR = 23.04, 95% C.I. = 9.74 -54.53, *p =* 9.49 × 10^−13^) (Figure 1B and Table 3).

**Table 3:** *HYAL2* methylation in Stage I&II PDAC comparing to controls

CpG sites	Controls median (IQR)	PDAC stage I&II median (IQR)	OR (95 % CI) * per −10% methylation	*p*-value *
**HYAL2_CpG_1**	0.35 (0.31-0.40)	0.25 (0.20-0.33)	3.17 (2.06-4.86)	**1.36E-07**
**HYAL2_CpG_2**	0.27 (0.23-0.31)	0.14 (0.09-0.19)	10.98 (5.76-20.92)	**3.19E-13**
**HYAL2_CpG_3**	0.43 (0.38-0.46)	0.28 (0.23-0.34)	23.04 (9.74-54.53)	**9.49E-13**
**HYAL2_CpG_4**	0.61 (0.56-0.66)	0.48 (0.43-0.55)	5.12 (3.15-8.27)	**3.55E-11**

* Logistic regression, adjusted for age and gender

### *HYAL2* methylation has no correlation with the clinical characteristics of PDAC

With the aim to further identify subgroups at risk for PDAC, the relation between blood-based *HYAL2* methylation status and clinical characteristics of PDAC (Table 1) was analysed. The methylation levels of all four CpG sites in HYAL2-A amplicon showed no correlation with tumor size, lymph node involvement, status of distant metastasis, tumor stage, grading as shown in Table 4. This gives further proof that *HYAL2* methylation could detect PDAC regardless of stage, and thus, is suitable for the early detection. We further evaluated the influence of age and gender to *HYAL2* methylation. Interestingly, *HYAL2* methylation showed a significant inverse correlation with older age and a positive correlation with male gender in the control group but not in the case group (Table 5).

**Table 4:** The methylation of *HYAL2* in PDAC patients with different clinical characteristics

Clinical characteristics (N)	Group (N)	Median of age	Median of methylation levels			
			HYAL2_CpG_1	HYAL2_CpG_2	HYAL2_CpG_3	HYAL2_CpG_4
**Tumour size (70)**	< pT3 (4)	64.20	0.27	0.16	0.24	0.46
	pT3 & pT4 (66)	62.64	0.25	0.14	0.26	0.48
	*p*-value *	0.544	0.839	0.693	0.770	0.444
**Lymph node involvement (70)**	pN0 (16)	61.51	0.27	0.12	0.26	0.47
	pN1 (54)	63.56	0.25	0.14	0.28	0.48
	*p*-value *	0.611	0.279	0.249	0.788	0.725
**Status of distant metastasis (78)**	M0 (66)	62.73	0.25	0.14	0.26	0.48
	M1 (12)	69.09	0.30	0.13	0.26	0.45
	*p*-value *	0.144	0.267	0.811	0.822	0.779
**Tumour stage (77)**	Stage I&II (61)	63.00	0.25	0.14	0.28	0.48
	Stage III&IV (16)	65.50	0.27	0.16	0.24	0.45
	*p*-value *	0.899	0.407	0.939	0.403	0.888
**Grading (57)**	Grade 1&2 (35)	64.42	0.26	0.14	0.28	0.49
	Grade 3 (22)	60.50	0.23	0.15	0.24	0.46
	*p*-value *	0.422	0.133	0.693	0.111	0.083

**Table 5:** Correlation between *HYAL2* methylation and age and gender

**Correlation with older age**				
**CpG sites**	**Controls**		**Cases**	
	**Spearman rho**	***p*-value**	**Spearman rho**	***p*-value**
**HYAL2_CpG_1**	−0.159	**0.028**	0.042	0.711
**HYAL2_CpG_2**	−0.161	**0.026**	0.124	0.266
**HYAL2_CpG_3**	−0.150	**0.039**	0.088	0.431
**HYAL2_CpG_4**	−0.107	0.142	0.060	0.593
**Correlation with male gender**				
**CpG sites**	**Controls**		**Cases**	
	**Spearman rho**	***p*-value**	**Spearman rho**	***p*-value**
**HYAL2_CpG_1**	0.142	**0.049**	0.149	0.181
**HYAL2_CpG_2**	0.143	**0.048**	0.036	0.750
**HYAL2_CpG_3**	0.179	**0.013**	−0.051	0.652
**HYAL2_CpG_4**	0.160	**0.027**	−0.066	0.554

### *HYAL2* methylation has no correlation with the overall survival of PDAC

**Figure 2:**
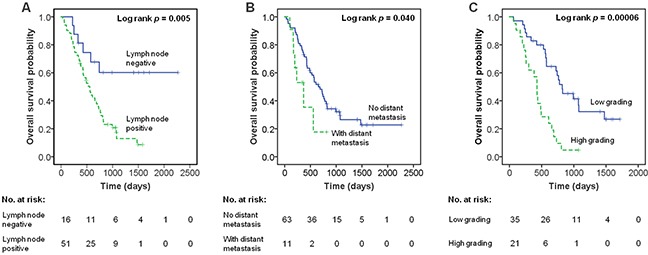
The association of clinical characteristic with overall survival of PDAC by Kaplan–Meier curves **(A)** Association of lymph node with overall survival of PDAC. **(B)** Association of distant metastatic status with overall survival of PDAC. **(C)** Association of grading with overall survival of PDAC. Low grading: grading 1 and 2; High grading: grading 3.

Among the 82 PDAC patients, 61 had complete follow up for their overall survival record for four years (stage I = 3, stage II = 47, stage III = 2, stage IV = 4). In the four years-time period, 8 PDAC patients were still alive (all 3 Stage I PDAC patients, 3 out of all 9 Stage IIA patients, and 2 out of all 38 Stage IIB patients). 53 PDAC patients died with a median overall survival time of 423 days, range from 32 days to 1475 days. The cox regression analysis was performed to evaluate the influence of individual factors to the overall survival of PDAC patients (tumor size was not analysed in the cox regression, because no statistical power can be reached when only 4 patients had T1 and T2 tumors). In the univariate analysis, positive lymph nodes, status of distant metastasis, and higher grading showed significant influence on the survival time (*p* < 0.05), whereas gender, age and *HYAL2* methylation levels had no significant correlation with the overall survival of PDAC patients (Table 6). In Figure 2, Kaplan Meier curve also shows that the overall survival time is significantly correlated with the status of lymph nodes, status of distant metastasis and grading. In the following multivariate analysis using the significant factors (*p* < 0.1) selected from the univariate model, status of distant metastasis and grading correlated significantly with the overall survival of PDAC patients (status of distant metastasis, hazard ratio = 5.97, *p* = 0.006; higher grading, hazard ratio = 3.52, *p* = 0.0003, Table 6).

**Table 6:** Prognostic factors of overall survival in PDAC patients: result from cox regression model

Factors	Total event (N)	Univariate		Multivariate	
		HR (95 % CI)	*p*-value	HR (95 % CI)	*p*-value
**Positive lymph node**	48	3.22 (1.36-7.65)	**0.008**	1.77 (0.66-4.72)	0.253
**Distant metastasis**	51	2.31 (1.01-5.28)	**0.046**	5.97 (1.66-21.47)	**0.006**
**Higher grading**	42	3.52 (1.84-6.75)	**0.0001**	3.52 (1.79-6.93)	**0.0003**
**Male gender**	53	1.20 (0.69-2.09)	0.514	-	-
**Older age** *	53	1.00 (0.58-1.71)	0.985	-	-
**Lower HYAL2_CpG_1 ^§^**	53	1.05 (0.61-1.80)	0.866	-	-
**Lower HYAL2_CpG_2 ^§^**	53	0.95 (0.55-1.64)	0.840	-	-
**Lower HYAL2_CpG_3 ^§^**	53	1.16 (0.67-2.00)	0.590	-	-
**Lower HYAL2_CpG_4 ^§^**	53	0.91 (0.53-1.58)	0.745	-	-

* Age older than or equal to the medium age of the PDAC patients

^§^ Methylation level lower than the medium methylation levels of the PDAC patients

### *HYAL2* methylation as marker for the detection of PDAC

The possibility of using *HYAL2* methylation as detection marker of PDAC was evaluated with the receiver operating characteristic [26] curve analyses adjusted for age and gender by logistic regression. The methylation levels of all *HYAL2* methylation sites pooled together revealed strong discriminatory power between PDAC cases and controls (area under curve (AUC = 0.92, 95% C.I. = 0.88-0.96, sensitivity 75.6%, specificity 93.7%), and between stage I&II PDAC cases and controls (AUC = 0.93, 95% C.I. = 0.89-0.98, sensitivity 66.7%, specificity 95.3%) (Table 7). Among the four HYAL2 CpG sites, HYAL2_CpG_2 and HYAL2_CpG_3 CpG sites contributed the most distinguish power, and each of the CpG sites could distinguish all the PDAC cases and the I&II PDAC cases from controls with AUC > 0.90 as shown in Table 7.

**Table 7:** The discriminatory power of methylation in *HYAL2* to distinguish PDAC cases from healthy controls

**All 82 PDAC cases vs. all 191 controls**			
**CpG sites**	**AUC, 95% CI**	**Sensitivity (positive predictive value)**	**Specificity (negative predictive rate)**
**HYAL2_CpG_1**	0.74 (0.66-0.81)	37.80%	98.40%
**HYAL2_CpG_2**	0.90 (0.85-0.95)	73.20%	94.20%
**HYAL2_CpG_3**	0.92 (0.88-0.96)	74.40%	94.80%
**HYAL2_CpG_4**	0.85 (0.80-0.91)	59.80%	92.70%
**All HYAL2 CpG sites**	0.92 (0.88-0.96)	75.60%	93.70%
**60 PDAC cases of Stage I&II vs. all 191 controls**			
**CpG sites**	**AUC, 95% CI**	**Sensitivity (positive predictive value)**	**Specificity (negative predictive rate)**
**HYAL2_CpG_1**	0.77 (0.68-0.85)	41.70%	98.40%
**HYAL2_CpG_2**	0.90 (0.84-0.95)	68.30%	94.20%
**HYAL2_CpG_3**	0.93 (0.89-0.97)	68.30%	94.80%
**HYAL2_CpG_4**	0.85 (0.79-0.91)	53.30%	94.80%
**All HYAL2 CpG sites**	**0.93 (0.89-0.98)**	66.70%	95.30%

* Logistic regression, adjusted for age and gender

## DISCUSSION

This is the first case-control study that describes a significant correlation between decreased methylation of HYAL2 in peripheral blood among PDAC patients compared to healthy controls. PDAC remains a highly lethal cancer, with about 80% of the patients diagnosed at advanced and unresectable stage (20% are at stage III, median survival 8-14 months; 60% are at stage IV, median survival 4-6 months). The other resectable patients (stage 0, stage I, stage II and even part stage III) have a median survival of about 20 months after the surgery [27]. In our study, we also observed that the early detection of PDAC could dramatically increase the survival time of patients (in our study, 100% of Stage I, 33.3% of Stage IIA and 5% of Stage IIB patients survived for more than four years). The detection of PDAC at resectable stage (before and including stage II) can improve survival time of patients for more than 10 months; whereas clinicians are devoted to improve 2-3 months’ survival of advanced PDAC patients in many Phase III clinical studies [28–29]. Early detection markers for resectable pancreatic neoplasia are urgently needed. However, the carbohydrate antigen (CA) 19-9, the tumor marker of pancreatic cancer, is related to tumor size and has a limited sensitivity for small cancers [30]. The positive predictive value of CA 19-9 is especially low in asymptomatic individuals and is therefore not recommended as screening tool [31]. Genetic analysis found several PDAC specific genes and identifies individuals with a germline mutation associated with predispositions for familial cancers. Among pancreatic cancer patients 3-16% are either syndromic or familial [32]. Therefore, diagnostic tools are urgently needed to provide surveillance strategies for predisposed carriers [32]. Additionally, a sub-classification of gene mutation carriers with an increased risk of developing pancreatic cancer is desirable to identify especially those people at higher risk, in order to develop more individualized surveillance programs.

*HYAL2* is known to be increased in proliferative processes and was described as a tumor suppressor gene involved in cell adhesion, cell mobility, chemokinesis, cancer progression, angiogenesis and metastasis [21, 33–37]. Recently, we have shown lower *HYAL2* methylation level and higher HYAL2 gene expression in the peripheral blood of breast cancer patients compared to controls [25]. This is in agreement with previous studies publishing decreased *HYAL2* methylation in the peripheral blood in the patients with head and neck squamous cell carcinoma compared to controls [11]. Here, we report *HYAL2* hypomethylation in the blood of PDAC patients comparing to the healthy controls. It is possible that hypomethylation of *HYAL2* may reflect a broader change in DNA methylation across many CpG sites in the genome that occurs in patients with certain cancer syndromes. But it is interesting that CpG_3 of *HYAL2* showed the most significant correlation to PDAC, whereas CpG_4 of *HYAL2* was the most significant site correlated to breast cancer. Studies with larger samples size and multiple cancers will be helpful to understand whether there are cancer type specific methylation patterns. It has been known that the proportion of DNA from cancer cells in blood has a ratio of about 1:1000 comparing to the DNA from blood cells [38], and thus, very likely the change of DNA methylation mainly originates from white blood cells. In our previous study in breast cancer, we suggested that the change of leucocytes subpopulation proportion and the breast cancer associated differential *HYAL2* methylation in leucocytes subpopulations (T cells and probably other cell types except B cells) are the main reasons for the origin of breast cancer associated differential methylation in blood [25]. Unfortunately, in our study we only have DNA materials from the whole blood of the PDAC patients and controls, and no fresh blood samples available; therefore we could not tell whether this altered DNA methylation is because of the change in proportion of cells and methylation of this gene is cell type specific. Another way to estimate the cell proportion is via statistical calculation using epigenome-wide Array data, which is also not available for us. This is a limitation of our study, which should be further explored in the future. In addition, as no questionnaires to the life style were given to the patients and the blood donors, there was in lack of details to the life style factors of the patients and healthy controls. Except age and gender, other potential methylation related factors, such as tobacco smoking and alcohol drinking are unfortunately not available in our study. In future studies these clinical and life style data of study subjects should be collected.

The mechanisms of cancer-related aberrant *HYAL2* methylation in blood remain unknown. So far, there is also no report about the function of HYAL2 in blood cells. Very recently, copy number variation in the gene region of *HYAL2* was reported to be associated with diffuse large B-cell lymphoma [23], indicating that *HYAL2* is functional in the malignancy of B cells. In our study, we disclosed the highly significant difference of *HYAL2* methylation between PDAC cases and controls, and suggested its potential in the clinical usage as a marker for the PDAC. We could not find a correlation between the altered *HYAL2* methylation in blood cells and the clinical pathological characteristics. The similar HYAL2 methylation levels in the patients with early and advance PDAC, as well as the comparable distinguish power for *HYAL2* methylation for the PDAC in general and stage I&II PDAC, give strong indication that blood-based *HYAL2* methylation might be sufficient for the detection of resectable PDAC. Our finding of HYLA2 hypomethylation in the blood of PDAC patients by case-control study cannot answer the question of whether methylation marker presents cancer risk or cancer progress. In a prospective cass-cohort analysis of DNA methylation in blood and breast cancer, Xu and colleagues found the change of methylation in women whose blood sample was collected less than 1 year before diagnosis is more pronounced than in women who provided blood in the year before their diagnosis. Although Xu's study indicates that blood-based methylation might be marker for preclinical disease via showing the correlation between marker strength and the time to diagnosis, the hypothesis should be taken with caution due to limited sample size. More prospective case-cohort studies with larger sample size will be needed to explore whether methylation is a marker of predisposition to disease or a marker of preclinical disease. In the future studies, it will be interesting to analyse other potential markers. The combination of *HYAL2* methylation and other markers, especially the markers representing different pathway or mechanism, might provide a even better panel for the detection of early PDAC, and might even be useful for sub-classification and prognosis [22].

To highlight, this study disclosed the correlation between altered DNA methylation in whole blood (mainly from blood cells) and PDAC, and suggested outstanding power for the detection of PDAC, and has great potential for the detection of early PDAC. In contrast, so far circulating free DNA methylation markers from serum or plasma has shown limited evidence for the clinical usage of PDAC detection due to limited sensitivity to the early stage tumors [39]. Although blood-based *HYAL2* methylation has shown significant difference between PDAC patients and controls in our study, the power of this study is limited by the small sample size. An additional remark also for our study is that the methylation difference has been compared to healthy controls but not to non-malignant pancreatic diseases or other cancers. Validation studies in a second cohort with larger sample sizes and even prospective studies among high risk persons are needed to address the effect of predictive potential in healthy subjects.

## MATERIALS AND METHODS

### Study population

The present study was approved by the local Ethics Committee of the University of Heidelberg, Germany. Each participant provided written informed consent. All participants were Caucasian. Blood sample was drawn by venipuncture, collected into Li-Heparin-Gel Monovette (S-Monovette® 7.5ml LH; cat# 01.1604.001; Sarstedt AG; Nümbrecht, Germany) and stored at −80oC until DNA isolation. The DNA is extracted by the QIAamp DNA Blood Mini Kit (Cat No./ID: 51104) according to manufacturer's instructions.

Peripheral blood samples were obtained from 82 PDAC patients (stage I = 4, stage II = 57, stage III = 4, stage IV = 12, stage unknown = 5) at the University of Heidelberg University Hospital of Surgery in Heidelberg from September of 2009 to August of 2011. The PDAC patients have a median age of 63.7 years old (range from 39 to 79 years old), and have 49 males (59.8%) and 33 females (40.2%). Since the PDAC patients recruited in this study are part of a surgical collective, more early and resectable cases (Stage I&II) were obtained than typical for the general PDAC population. In PDAC surgery, if metastases are found, the operation is finished as exploration without resection but with histologic confirmation. Thus, several Stage III and Stage IV cases are also included in the study. The clinical characteristics of PDAC were confirmed by pathology in all cases. All blood samples from the enrolled PDAC patients were taken before the surgery and pancreatic cancer related treatment. Detailed clinical data of the PDAC patients are shown in Table 3. In the follow up period, 21 PDAC patients had no or not complete follow up data, whereas the remaining 61 PDAC patients were followed for four years with 53 death event in this period. No progression free survival data is available for the PDCA cases.

Peripheral blood samples from controls were consecutively collected from healthy blood donors (N = 191) by the German Red Cross Blood Service of Baden-Württemberg-Hessen (Mannheim, Germany) from 2004 to 2012 as described elsewhere [25, 40]. The controls have a median age of 61.0 years old (range from 21 to 68 years old), and have 115 males (60.2%) and 76 females (39.8%). No further exclusion or inclusion criteria were applied for the controls.

### MALDI-TOF mass spectrometry

MALDI-TOF mass spectrometry (Sequenom) described by Breitling *et al*. [9, 41] was used in all the validation and further exploring rounds. In brief, DNA was bisulfite converted by EZ-96 DNA Methylation Gold Kit (Zymo Research) and amplified by bisulfite-specific primers (no SNPs in the primers) and PCR amplicons HYAL2-A were described as previously used [25]. The PCR products were used according to the standard protocol of Sequenom EpiTyper Assay, and further cleaned by Resin and dispensed to a 384 SpectroCHIP by a Nanodispenser as described by us before. The chips were read by a Sequenom Mass Spectrometer system. Data were collected by SpectroACQUIRE v3.3.1.3 software and visualized with MassArray EpiTyper v1.2 software. For each batch of MassARRAY analysis, same amount of cases and controls were randomly selected from the cohort [25].

### Statistical analyses

All the statistical analyses were conducted by SPSS Statistics 21. Spearman's rank correlation coefficient was used to assess the correlations. The comparisons between two and multiple groups were performed by logistic regression models and non-parametric tests. The logistic regression results were adjusted for possible and available confounding effects such as age and gender by including additional co-variables into the logistic regression models. Receiver operating characteristic [26] curve analysis assessed the discriminatory power of methylation levels. Cox-regression analyses were used to estimate the influence of factors to the survival of PDAC patients. All statistical tests were two-sided, and *p* values < 0.05 were defined as statistically significant.

## Abbreviations

AUC: area under curve
CA: carbohydrate antigen
CI: confidence interval
HYAL2: hyaluronoglucosaminidase 2 gene
HR: hazard ratio
IQR: inter quartile range
MALDI-TOF: matrix-assisted laser desorption ionization time-of-flight
PDAC: pancreatic ductal adenocarcinoma
OR: odds ratio

## References

[1] Hackert T, Tjaden C, Muller S, Hinz U, Hartwig W, Strobel O, Fritz S, Schmied B, Buchler MW, Werner J (2012). Extrapancreatic malignancies in patients with pancreatic cancer: epidemiology and clinical consequences. Pancreas.

[2] Ruess DA, Makowiec F, Chikhladze S, Sick O, Riediger H, Hopt UT, Wittel UA (2015). The prognostic influence of intrapancreatic tumor location on survival after resection of pancreatic ductal adenocarcinoma. BMC Surg.

[3] Conroy T, Desseigne F, Ychou M, Bouche O, Guimbaud R, Becouarn Y, Adenis A, Raoul JL, Gourgou-Bourgade S, de la Fouchardiere C, Bennouna J, Bachet JB, Khemissa-Akouz F (2011). FOLFIRINOX versus gemcitabine for metastatic pancreatic cancer. N Engl J Med.

[4] (2016). http://www.cancerresearchuk.org/health-professional/cancer-statistics/statistics-by-cancer-type/pancreatic-cancer/survival#heading-Zero.

[5] Ghiorzo P (2014). Genetic predisposition to pancreatic cancer. World J Gastroenterol.

[6] Holter S, Borgida A, Dodd A, Grant R, Semotiuk K, Hedley D, Dhani N, Narod S, Akbari M, Moore M, Gallinger S (2015). Germline BRCA mutations in a large clinic-based cohort of patients with pancreatic adenocarcinoma. J Clin Oncol.

[7] Weber M, Davies JJ, Wittig D, Oakeley EJ, Haase M, Lam WL, Schubeler D (2005). Chromosome-wide and promoter-specific analyses identify sites of differential DNA methylation in normal and transformed human cells. Nat Genet.

[8] Feinberg AP, Tycko B (2004). The history of cancer epigenetics. Nat Rev Cancer.

[9] Breitling LP, Yang R, Korn B, Burwinkel B, Brenner H (2011). Tobacco-smoking-related differential DNA methylation: 27K discovery and replication. Am J Hum Genet.

[10] Heyn H, Carmona FJ, Gomez A, Ferreira HJ, Bell JT, Sayols S, Ward K, Stefansson OA, Moran S, Sandoval J, Eyfjord JE, Spector TD, Esteller M (2013). DNA methylation profiling in breast cancer discordant identical twins identifies DOK7 as novel epigenetic biomarker. Carcinogenesis.

[11] Langevin SM, Koestler DC, Christensen BC, Butler RA, Wiencke JK, Nelson HH, Houseman EA, Marsit CJ, Kelsey KT (2012). Peripheral blood DNA methylation profiles are indicative of head and neck squamous cell carcinoma: an epigenome-wide association study. Epigenetics.

[12] Marsit CJ, Koestler DC, Christensen BC, Karagas MR, Houseman EA, Kelsey KT (2011). DNA methylation array analysis identifies profiles of blood-derived DNA methylation associated with bladder cancer. J Clin Oncol.

[13] Teschendorff AE, Menon U, Gentry-Maharaj A, Ramus SJ, Gayther SA, Apostolidou S, Jones A, Lechner M, Beck S, Jacobs IJ, Widschwendter M (2009). An epigenetic signature in peripheral blood predicts active ovarian cancer. PLoS One.

[14] Brennan K, Garcia-Closas M, Orr N, Fletcher O, Jones M, Ashworth A, Swerdlow A, Thorne H, Riboli E, Vineis P, Dorronsoro M, Clavel-Chapelon F, Panico S (2012). Intragenic ATM methylation in peripheral blood DNA as a biomarker of breast cancer risk. Cancer Res.

[15] Flanagan JM, Munoz-Alegre M, Henderson S, Tang T, Sun P, Johnson N, Fletcher O, Dos Santos Silva I, Peto J, Boshoff C, Narod S, Petronis A (2009). Gene-body hypermethylation of ATM in peripheral blood DNA of bilateral breast cancer patients. Hum Mol Genet.

[16] Iwamoto T, Yamamoto N, Taguchi T, Tamaki Y, Noguchi S (2011). BRCA1 promoter methylation in peripheral blood cells is associated with increased risk of breast cancer with BRCA1 promoter methylation. Breast Cancer Res Treat.

[17] Widschwendter M, Apostolidou S, Raum E, Rothenbacher D, Fiegl H, Menon U, Stegmaier C, Jacobs IJ, Brenner H (2008). Epigenotyping in peripheral blood cell DNA and breast cancer risk: a proof of principle study. PLoS One.

[18] Maierthaler M, Kriegsmann M, Peng C, Jauch S, Szabo A, Wallwiener M, Rom J, Sohn C, Schneeweiss A, Sinn HP, Yang R, Burwinkel B (2015). S100P and HYAL2 as prognostic markers for patients with triple-negative breast cancer. Exp Mol Pathol.

[19] Wu M, Cao M, He Y, Liu Y, Yang C, Du Y, Wang W, Gao F (2015). A novel role of low molecular weight hyaluronan in breast cancer metastasis. FASEB J.

[20] Tang Q, Holland-Letz T, Slynko A, Cuk K, Marme F, Schott S, Heil J, Qu B, Golatta M, Bewerunge-Hudler M, Sutter C, Surowy H, Wappenschmidt B (2016). DNA methylation array analysis identifies breast cancer associated – RPTOR, MGRN1 and RAPSN hypomethylation in peripheral blood DNA. Oncotarget.

[21] Chowdhury B, Hemming R, Faiyaz S, Triggs-Raine B (2016). Hyaluronidase 2 (HYAL2) is expressed in endothelial cells, as well as some specialized epithelial cells, and is required for normal hyaluronan catabolism. Histochem Cell Biol.

[22] Nykopp TK, Pasonen-Seppanen S, Tammi MI, Tammi RH, Kosma VM, Anttila M, Sironen R (2015). Decreased hyaluronidase 1 expression is associated with early disease recurrence in human endometrial cancer. Gynecol Oncol.

[23] Mareschal S, Dubois S, Viailly PJ, Bertrand P, Bohers E, Maingonnat C, Jais JP, Tesson B, Ruminy P, Peyrouze P, Copie-Bergman C, Fest T, Jo Molina T (2016). Whole exome sequencing of relapsed/refractory patients expands the repertoire of somatic mutations in diffuse large B-cell lymphoma. Genes Chromosomes Cancer.

[24] Pfutze K, Benner A, Hoffmeister M, Jansen L, Yang R, Blaker H, Herpel E, Ulrich A, Ulrich CM, Chang-Claude J, Brenner H, Burwinkel B (2015). Methylation status at HYAL2 predicts overall and progression-free survival of colon cancer patients under 5-FU chemotherapy. Genomics.

[25] Yang R, Pfutze K, Zucknick M, Sutter C, Wappenschmidt B, Marme F, Qu B, Cuk K, Engel C, Schott S, Schneeweiss A, Brenner H, Claus R (2015). DNA methylation array analyses identified breast cancer-associated HYAL2 methylation in peripheral blood. Int J Cancer.

[26] Pickartz T, Ringel F, Wedde M, Renz H, Klein A, von Neuhoff N, Dreger P, Kreuzer KA, Schmidt CA, Srock S, Schoeler D, Schriever F (2001). Selection of B-cell chronic lymphocytic leukemia cell variants by therapy with anti-CD20 monoclonal antibody rituximab. Exp Hematol.

[27] Vincent A, Herman J, Schulick R, Hruban RH, Goggins M (2011). Pancreatic cancer. Lancet.

[28] Gill S, Ko YJ, Cripps C, Beaudoin A, Dhesy-Thind S, Zulfiqar M, Zalewski P, Do T, Cano P, Lam WY, Dowden S, Grassin H, Stewart J (2016). PANCREOX: a randomized phase III study of 5-fluorouracil/leucovorin with or without oxaliplatin for second-line advanced pancreatic cancer in patients who have received gemcitabine-based chemotherapy. J Clin Oncol.

[29] Wang-Gillam A, Li CP, Bodoky G, Dean A, Shan YS, Jameson G, Macarulla T, Lee KH, Cunningham D, Blanc JF, Hubner RA, Chiu CF, Schwartsmann G (2016). Nanoliposomal irinotecan with fluorouracil and folinic acid in metastatic pancreatic cancer after previous gemcitabine-based therapy (NAPOLI-1): a global, randomised, open-label, phase 3 trial. Lancet.

[30] Cwik G, Wallner G, Skoczylas T, Ciechanski A, Zinkiewicz K (2006). Cancer antigens 19-9 and 125 in the differential diagnosis of pancreatic mass lesions. Arch Surg.

[31] Kim JE, Lee KT, Lee JK, Paik SW, Rhee JC, Choi KW (2004). Clinical usefulness of carbohydrate antigen 19-9 as a screening test for pancreatic cancer in an asymptomatic population. J Gastroenterol Hepatol.

[32] Klein AP, Hruban RH, Brune KA, Petersen GM, Goggins M (2001). Familial pancreatic cancer. Cancer J.

[33] Afratis N, Gialeli C, Nikitovic D, Tsegenidis T, Karousou E, Theocharis AD, Pavao MS, Tzanakakis GN, Karamanos NK (2012). Glycosaminoglycans: key players in cancer cell biology and treatment. FEBS J.

[34] Duterme C, Mertens-Strijthagen J, Tammi M, Flamion B (2009). Two novel functions of hyaluronidase-2 (Hyal2) are formation of the glycocalyx and control of CD44-ERM interactions. J Biol Chem.

[35] Hesson LB, Cooper WN, Latif F (2007). Evaluation of the 3p21.3 tumour-suppressor gene cluster. Oncogene.

[36] Saito T, Kawana H, Azuma K, Toyoda A, Fujita H, Kitagawa M, Harigaya K (2011). Fragmented hyaluronan is an autocrine chemokinetic motility factor supported by the HAS2-HYAL2/CD44 system on the plasma membrane. Int J Oncol.

[37] Wang F, Grigorieva EV, Li J, Senchenko VN, Pavlova TV, Anedchenko EA, Kudryavtseva AV, Tsimanis A, Angeloni D, Lerman MI, Kashuba VI, Klein G, Zabarovsky ER (2008). HYAL1 and HYAL2 inhibit tumour growth in vivo but not in vitro. PLoS One.

[38] Hoque MO, Feng Q, Toure P, Dem A, Critchlow CW, Hawes SE, Wood T, Jeronimo C, Rosenbaum E, Stern J, Yu M, Trink B, Kiviat NB (2006). Detection of aberrant methylation of four genes in plasma DNA for the detection of breast cancer. J Clin Oncol.

[39] Henriksen SD, Madsen PH, Krarup H, Thorlacius-Ussing O (2015). DNA hypermethylation as a blood-based marker for pancreatic cancer: a literature review. Pancreas.

[40] Yang R, Schlehe B, Hemminki K, Sutter C, Bugert P, Wappenschmidt B, Volkmann J, Varon R, Weber BH, Niederacher D, Arnold N, Meindl A, Bartram CR (2010). A genetic variant in the pre-miR-27a oncogene is associated with a reduced familial breast cancer risk. Breast Cancer Res Treat.

[41] Breitling LP, Salzmann K, Rothenbacher D, Burwinkel B, Brenner H (2012). Smoking, F2RL3 methylation, and prognosis in stable coronary heart disease. Eur Heart J.

